# Evaluation of Total Homocysteine Levels in Relation to Abdominal Fat Mass and Traditional Cardiovascular Risk Factors in Overweight and Obese Adolescents

**DOI:** 10.3390/life15081329

**Published:** 2025-08-21

**Authors:** Małgorzata Rumińska, Ewelina Witkowska-Sędek, Maria Krajewska, Anna Stelmaszczyk-Emmel, Maria Sobol, Beata Pyrżak

**Affiliations:** 1 Department of Pediatrics and Endocrinology, Medical University of Warsaw, 02-091 Warsaw, Poland; 2 Department of Laboratory Diagnostics and Clinical Immunology of Developmental Age, Medical University of Warsaw, 02-091 Warsaw, Poland; 3 Department of Biophysics, Physiology and Pathophysiology, Medical University of Warsaw, 02-091 Warsaw, Poland

**Keywords:** homocysteine, cardiovascular risk factors, obesity, adolescents

## Abstract

Cardiovascular diseases remain the leading cause of mortality worldwide, with multiple risk factors contributing to their development. Among these, obesity and hyperhomocysteinemia have been recognized as significant contributors to endothelial dysfunction, a key early event in the pathogenesis of atherosclerosis. Our study aimed to evaluate the relationship between total homocysteine (tHcy) levels and traditional cardiovascular risk factors in overweight and obese adolescents. We enrolled 42 obese, 14 overweight, and 25 non-obese children. No significant differences in tHcy levels were observed between overweight, obese, and non-obese adolescents. Homocysteine positively correlated with age (r = 0.433, *p* < 0.011) and creatinine concentrations (r = 0.363, *p* = 0.001) in the overall group of overweight, obese, and non-obese children, as well as in the combined group of overweight and obese children (for age: r = 0.275, *p* = 0.025; for creatinine: r = 0.278, *p* = 0.025). We did not find any association between homocysteine and atherogenic lipid profile, insulin-resistance status, blood pressure, and inflammatory parameters in overweight and obese patients. Age emerged as the strongest independent predictor of homocysteine levels. The observed association with creatine suggests a potential renal contribution to homocysteine metabolism.

## 1. Introduction

Elevated homocysteine (Hcy) levels are recognized as an independent risk factor for cardiovascular disease (CVD) [1,2]. Homocysteine, a sulfur-containing amino acid, is produced as an intermediate during the demethylation of methionine, an essential amino acid obtained primarily from dietary sources, especially meat. Its metabolism is regulated by vitamin-derived cofactors, particularly vitamins B6, B2, B12, and folate (vitamin B9), which facilitate either the remethylation of Hcy back to methionine or its conversion via transsulfuration to cysteine. Several factors influence Hcy levels, including age, smoking, high coffee consumption, certain drugs, as well as genetic, epigenetic, and nutritional factors [3,4,5,6,7].

Hyperhomocysteinemia (HHcy) contributes to mitochondrial dysfunction and the acceleration of cellular senescence. Moreover, it induces oxidative stress and vascular inflammation, stimulates the expression of interleukins and chemokines, increases intracellular accumulation of reactive oxygen species (ROS), reduces nitric oxide (NO) bioavailability, causes hypomethylation of deoxyribonucleic acid (DNA), ribonucleic acid (RNA), and proteins in endothelial cells, promotes protein homocysteinylation, and increases smooth muscle cell proliferation. Together, these effects weaken endothelial function and raise cardiovascular risk [5,8]. In addition to its well-established association with cerebrovascular and cardiovascular disorders, HHcy has been linked to the development of several other conditions, such as neurodegenerative diseases, fatty liver diseases, thrombotic events, chronic kidney disease (CKD), rheumatoid arthritis, eye disorders, osteoporosis, and various malignancies [1,5,8,9,10,11,12].

Considering the role of HHcy in the pathogenesis of atherosclerosis and the significant global impact of obesity on CVD development, an important and still not fully elucidated question arises: is there a link between excess fat mass and HHcy-related disturbances that may exacerbate the health risks associated with obesity?

Recent research suggests that obesity may contribute to HHcy through several mechanisms. Chronic low-grade inflammation and oxidative stress associated with obesity may disrupt Hcy metabolism. Additionally, obese individuals often have poor dietary habits, leading to deficiencies in key vitamins necessary for Hcy breakdown. Insulin resistance (IR), a common complication of obesity, is known to impair the enzymatic pathways involved in Hcy clearance [13,14]. In a meta-analysis [15] of 14 studies, researchers demonstrated remarkably higher Hcy levels in obese patients aged 22 to 61 years compared to non-obese individuals, regardless of nutritional status, dietary habits, IR status, presence of special disease, medication history, or genetic background. However, elevated Hcy levels were not dependent on the severity of obesity [15]. Other studies reported a link between HHcy and IR, diabetes, hyperlipidemia, and hypertension [8,9].

In the pediatric population, a systematic review and meta-analysis [16,17] showed that obese children and adolescents had higher Hcy levels compared to their non-obese counterparts. However, available data on the relationship between Hcy and abdominal adiposity, alterations in glucose and lipid profiles, IR, or blood pressure (BP) remain inconsistent [16,17,18,19,20,21,22,23,24,25,26,27,28,29,30,31]. With this in mind, our study aimed to evaluate the association between Hcy levels, excess fat mass, metabolic and inflammatory parameters, and BP in overweight and obese adolescents. Understanding the interplay between fat mass and HHcy may provide valuable insights into the mechanisms driving cardiovascular risk in this population.

## 2. Materials and Methods

This study was conducted at the Department of Pediatrics and Endocrinology, Medical University of Warsaw. A total of 56 adolescents aged 10 to 18 years were included: 42 obese (23 girls and 19 boys) and 14 overweight (4 girls and 10 boys). All of them were hospitalized in our unit due to excessive adiposity. The study group was compared with 25 healthy, non-obese, age- and sex-matched controls (14 girls and 11 boys).

All participants underwent a detailed medical history assessment and physical examination upon admission. Nutritional status was evaluated using BMI SDS (Body Mass Index Standard Deviation Score). Participants were classified as overweight if their BMI SDS was ≥+1, and as obese if their BMI SDS was ≥+2. Non-obese participants had a BMI SDS between −1.0 and +1.0 [32].

In the overweight and obese groups, secondary causes of excess adiposity, including genetic and endocrine disorders, were excluded. All enrolled adolescents did not have a diagnosis of diabetes mellitus, arterial hypertension, hepatic and renal disease, or any absorption disorders. They adhered to a standard, unrestricted diet—individuals following vegetarian or vegan diets were excluded from the study. Moreover, none of the participants received vitamin B12 or folic acid supplementation. Erythroid indices, including mean corpuscular volume (MCV), fell within the normal reference ranges. Family history was negative for both genetic and chronic conditions.

The study protocol was approved by the Bioethics Committee of the Medical University of Warsaw (approval no KB/272/2023).

### 2.1. Anthropometric Measurements

All adolescents’ anthropometric measurements were taken twice, and the results were averaged. Body weight was measured using a medical scale to the nearest 0.1 kg. Height was measured on a Holstein stadiometer to the nearest 0.1 cm. Measurements of waist (WC) and hip (HC) circumferences (cm), according to WHO recommendations, were used to calculate the waist-to-hip ratio (WHR) and the waist-to-height ratio (WHtR) [32]. Based on the thickness of skinfolds (mm) measured under the triceps brachii muscle and the inferior scapular angle, body fat mass percentage (%BFM ) was calculated using the Slaughter equations [33]. The BMI SDS was calculated using the least squares method, normalized for age and sex using Polish reference values [34,35].

### 2.2. Laboratory Tests

After 12 h of fasting, venous blood samples were collected. Serum total homocysteine (tHcy) levels (µmol/L) were determined using the enzyme immunoassay method (ELISA kits, EIA-2925, DRG Instruments GmbH, Marburg, Germany). Serum glucose concentrations (mg/dL); the lipid profile parameters of total cholesterol (TC, mg/dL), high-density lipoprotein cholesterol (HDL-C, mg/dL), and triglycerides (TG, mg/dL); and creatinine (mg/dL) were measured using standard enzymatic methods on the Vitros 5600 analyzer (Ortho Clinical Diagnostics, Raritan, NJ, USA). Low-density lipoprotein cholesterol (LDL-C, mg/dL) was calculated using Friedewald’s formula [36]. Insulin concentrations (μIU/mL) were determined using an immunoassay on the Immulite 2000 XPi analyzer (Siemens, Erlangen, Germany).

Complete blood counts were obtained using an automated blood cell counter (Sysmex XN 1000i hematological analyzer, Automated Hematology Analyzer, Sysmex XN -1000i, Sysmex Corporation, Kobe, Japan). C-reactive protein (CRP, mg/dL) concentrations were determined using a fixed-point immuno-rate method on the Vitros 5600 analyzer. An oral glucose tolerance test (OGTT) was conducted only in overweight and obese children.

The following atherogenic and insulin resistance indices were calculated: fasting glucose-to-insulin ratio (FGIR), Matsuda Index, Quantitative Insulin Sensitivity Check Index (QUICKI), Homeostasis Model Assessment—Insulin Resistance (HOMA-IR), triglyceride-to-high-density lipoprotein cholesterol ratio (TG/HDL-C), and non-HDL cholesterol [37,38,39,40]. We adopted the definition of HHcy as tHcy levels of ≥15 µmol/L [2,3].

### 2.3. Blood Pressure

We measured BP using an oscillometric device (Patient Monitor 6100, Philips Medizin Systeme Boeblingen GmBH, Boeblingen, Germany). The cuff size was adjusted to the child’s arm circumference, with cuff length and width corresponding to 80% and 40% of the arm circumference, respectively. Measurements were taken in a sitting position after 5–10 min of rest, with the child’s arm supported on a tabletop, back resting against the chair, and feet flat on the floor. Blood pressure was measured three times at 1–2 min intervals, and the average of these measurements was used for further analysis.

### 2.4. Statistical Methods

The Statistica program (version 13.3) was used for the analysis. The distribution of the obtained data was assessed using the Shapiro–Wilk test. Data with a normal distribution were presented as the mean ± standard deviation (SD), while non-parametric variables were expressed as the median (25th and 75th percentiles). We performed the analysis in non-obese, overweight, and obese children together, as well as in overweight and obese children both collectively and separately.

Depending on the distribution of variables, a Student’s *t*-test or Mann–Whitney U test was used to compare two groups, while a one-way analysis of variance (ANOVA) or Kruskal–Wallis test compared three groups. Post hoc tests were performed to identify which groups differed significantly. Correlation analyses utilized Spearman’s rank correlation coefficient. A multivariable linear regression analysis was used to assess the influence of selected anthropometric and laboratory parameters on serum tHcy levels. A significance level of *p* < 0.05 was deemed statistically significant for accepting or rejecting the statistical hypothesis.

## 3. Results

The anthropometric and laboratory characteristics of obese, overweight children, and the control group are presented in Table 1.

As anticipated, together with excess fat mass, a more insulin-resistant state and an atherogenic lipid profile were observed, with statistically significant differences between the obese and control groups. Obese adolescents exhibited significantly higher insulin concentrations, FGIR, and HOMA-IR, as well as a lower QUICKI, compared to the control group (*p* < 0.001 for all). In overweight children, these parameters showed a trend toward statistical significance (*p* = 0.080, *p* = 0.093, *p* = 0.074, and *p* = 0.052, respectively).

Furthermore, the obese group demonstrated decreased HDL-C levels (*p* = 0.006), increased TG concentrations (*p* < 0.001), and an elevated TG/HDL-C ratio (*p* < 0.001). In the overweight group, only TG concentrations and the TG/HDL-C ratio showed a tendency to differ from those of their non-obese peers (*p* = 0.061 and *p* = 0.077, respectively).

Among the remaining variables, systolic blood pressure (SBP) was significantly higher in the obese group compared to the control group (*p* = 0.015). Leukocyte profiles and CRP concentrations were comparable across all groups.

We did not observe significant differences in tHcy levels between these three groups. Similarly, no differences were found between girls and boys (the anthropometric and laboratory characteristics of girls and boys with overweight and obesity are presented in Table 2). In non-obese adolescents, plasma tHcy levels ranged from 7.36 µmol/L to 23.17 µmol/L, with elevated levels (tHcy ≥ 15 µmol/L) observed in six individuals (24%). Among overweight participants, tHcy levels ranged from 7.36 µmol/L to 14.15 µmol/L. In obese adolescents, tHcy levels ranged from 7.36 µmol/L to 23.17 µmol/L, with elevated levels (tHcy ≥ 15 µmol/L) detected only in five individuals (12%).

The Spearman correlation analysis revealed that tHcy positively correlated with age (r = 0.433, *p* < 0.011) and creatinine concentrations (r = 0.363, *p* = 0.001) in the overall group of overweight, obese, and non-obese adolescents analyzed together, as well as in the combined group of overweight and obese children (for age: r = 0.275, *p* = 0.025; for creatinine: r = 0.278, *p* = 0.025). The tendency to associate with age was also observed in separate analyses of overweight and obese children (r = 0.422, *p* = 0.091; r = 0.254, *p* = 0.078, respectively), along with a significant correlation with creatinine in the obese group (r = 0.304, *p* = 0.034). Additionally, in overweight children, tHcy correlated with WC (r = 0.547, *p* < 0.035). No statistically significant associations were found between tHcy and carbohydrate, lipid parameters, or inflammatory markers in both the overweight and obese children analyzed together and separately.

The models for multivariable backward regression analysis, which considered age, sex, BMI SDS, and WC as independent variables, were developed for all children, as well as for overweight and obese children, specifically for obese patients. In each group, age was the only variable found to have a statistically significant association with tHcy levels. The correlation coefficients were as follows: β = 0.408 ± 0.118 (95% CI: 0.172 to 0.643); β = 0.382 ± 0.136 (95% CI: 0.108 to 0.656); and β = 0.334 ± 0.162 (95% CI: 0.006 to 0.663), respectively. The models demonstrated statistical significance, with cumulative values of R^2^ = 0.166, *p* = 0.001; R^2^ = 0.146, *p* = 0.007; and R^2^ = 0.112, *p* = 0.046, respectively.

When we compared the anthropometric and biochemical parameters in a group of obese adolescents categorized by tHcy levels, individuals with tHcy ≥ 15 µmol/L were older than those with tHcy < 15 µmol/L (15.27 ± 2.45 vs. 13.67 ± 2.12, *p* < 0.001). No significant differences were found in BMI SDS [2.30 (2.20–2.60) vs. 2.30 (2.10–2.60), *p* = 0.922] or other anthropometric and biochemical parameters between these two subgroups. Similarly, in the control group, adolescents with HHcy were older than their peers with tHcy < 15 µmol/L (16.48 ± 0.65 vs. 14.51 ± 1.85, *p* = 0.018). As a result, they had slightly higher body weight [60.25 (58.50–69.50) vs. 54.00 (42.80–61.40), *p* = 0.060] and BMI [22.15 (2.90–23.20) vs. 18.70 (17.50–22.30), *p* = 0.086], but their BMI SDSs were comparable [0.40 (0.00–0.90) vs. 0.00 (−0.60–0.70), *p* = 0.390]. While in the subgroup of adolescents with HHcy, tHcy concentrations were statistically similar between those with normal weight and obese individuals [18.19 (16.32–18.99) vs. 16.60 (15.67–22.62), *p* = 1.00]. As expected, obese participants had significantly higher body weight [60.25 (58.50–69.50 vs. 95.00 (90.70–98.40) *p* = 0.008), BMI [22.15 (20.90–23.20) vs. 31.90 (30.20–33.70), *p* = 0.008), BMI SDS [0.40 (0.00–0.90) vs. 2.30 (2.20–2.60), *p* = 0.008), WC (68.00 ± 6.93 vs. 92.75 ± 9.53, *p* = 0.013), HC (89.66 ± 3.21 vs. 113.75 ± 9.60, *p* = 0.009), WHtR (0.41 ± 0.05 vs. 0.55 ± 0.02, *p* = 0.004), and %BFM (23.63 ± 8.61 vs. 36.92 ± 3.99, *p* = 0.039) compared to their normal-weight peers with HHcy. The normal-weight adolescents with HHcy tended to be slightly older than the obese individuals, but the difference was not statistically significant (16.48 ± 0.65 vs. 15.27 ± 2.45, *p* = 0.267).

## 4. Discussion

Our study did not find any effect of excess fat mass on serum tHcy levels, nor did it show an association with traditional cardiovascular risk factors usually linked to excess adiposity. Age was the only factor that influenced tHcy levels—both in the entire group and among overweight and obese children. Furthermore, obese adolescents with tHcy levels ≥ 15 µmol/L were older than their peers with lower levels. Data from a representative sample of children and adolescents in the United States (Third National Health and Nutrition Examination Survey, NHANES III [41]) indicated that Hcy levels were related to age and sex. Homocysteine levels increased with age, and higher values appeared in boys compared to girls around age 10. The generally higher Hcy levels seen in males may be explained by the stoichiometric production of Hcy during creatine/creatinine synthesis, a process proportional to muscle mass, which is typically greater in males. Additionally, it has been suggested that testosterone may downregulate the enzyme cystathionine β-synthase, thereby decreasing the conversion of Hcy to cysteine [42,43].

Growing evidence suggests that elevated Hcy levels may contribute to the development of obesity and IR. Both in vivo and in vitro studies have demonstrated that Hcy can inhibit lipolysis by activating adenosine monophosphate-activated protein kinase (AMPK) and its downstream target, acetyl-CoA carboxylase, thus promoting fat accumulation [44]. Furthermore, Hcy disrupts the insulin signaling by reducing insulin-stimulated tyrosine phosphorylation of the insulin receptor and insulin receptor substrate-1 (IRS-1), while increasing serine phosphorylation of IRS-1 and inhibiting Akt phosphorylation. These alterations collectively impair glucose uptake and contribute to metabolic dysfunction [8,14]. Impaired insulin signaling, particularly through the PI3K/Akt (phosphoinositide 3-kinase/protein kinase B) and MAPK (mitogen-activated protein kinase) pathways, leads to immune cell activation, ROS production, and the synthesis of pro-inflammatory cytokines via transcription factors such as NF-κB (nuclear factor kappa-light-chain-enhancer of activated B cells) [45]. Additionally, Hcy promotes systemic inflammation by stimulating the production of inflammatory cytokines: interleukin (IL)-1β, IL-6, IL-8, IL-18, tumor necrosis factor alpha (TNF-α), ROS, and resistin—an adipokine implicated in IR [5,8,46]. These changes, associated with obesity and linked to HHcy, contribute to the onset and progression of endothelial dysfunction [47].

In the meta-analysis by Leite et al. [16], which included six studies published between 1999 and 2017, three cross-sectional studies reported a positive but weak correlation between higher Hcy levels and overweight in children and adolescents, while the remaining cohort studies demonstrated a positive but statistically nonsignificant association. Moreover, the systematic review did not provide conclusive results regarding the relationship between Hcy and traditional risk factors, including abdominal obesity, lipid and glycemic profiles, IR, and BP [16].

The most recent meta-analysis by Ulloque-Badaracco et al. [17], encompassing 14 studies (4 case–control and 10 cross-sectional) published between 2004 and 2022, confirmed that children with obesity tend to exhibit elevated Hcy levels, despite comparable levels of vitamin B12 and folate between obese and non-obese groups. Notably, the authors emphasized substantial heterogeneity among study populations (age, sex, geographical origin, and definition of obesity) and the analytical methods used for Hcy determination. Among studies reporting higher Hcy levels in obese children compared to their non-obese counterparts, only a few examined the association with other metabolic or hemodynamic parameters related to overweight and obesity. For example, Abaci et al. [18], who uniquely applied the same standardized method (ELISA) for measuring tHcy as used in our study, investigated a cohort of 100 obese Turkish children (mean age, 10.2 ± 2.7 years) and found higher mean tHcy levels compared with controls (*p* = 0.041). They also reported positive correlations between tHcy and age, BMI, TG, and HDL-C, but no associations with TC, LDL-C, fasting glucose, insulin, or HOMA-IR. In another study, Kumar et al. [19] observed a significant association between Hcy and BMI in a cohort of 138 overweight and obese Indian children, but no correlations with plasma glucose or lipid profile parameters. In contrast, studies by Atabek et al. [20] and Narin et al. [21] found no associations between Hcy levels and age, BMI, total fat mass, lean body mass, WHR, fasting glucose and insulin, lipid profile, creatinine, vitamin B12, or BP in obese children. However, these studies reported independent associations between Hcy and folate [20] and leptin [21].

Deficiencies in folic acid, vitamin B12, and vitamin B6 contribute to elevated Hcy levels. Vitamin B6 functions as a coenzyme in the reaction where Hcy condenses with serine to form cystathionine, a process catalyzed by cystathionine β-synthase (CBS). Folic acid is involved in the remethylation of Hcy to methionine. In this pathway, Hcy accepts a methyl group derived from the conversion of 5-methyltetrahydrofolate (5-MTHF) to tetrahydrofolate (THF), or from the conversion of betaine to N,N-dimethylglycine (DMG). This remethylation process requires vitamin B12 as a cofactor and is catalyzed by methionine synthase (MS) [7,17].

In the meta-analysis mentioned above [17], only four studies failed to demonstrate a significant association between obesity and Hcy levels. Additionally, four studies not included in that meta-analysis [22,23,32,47] also reported no significant differences in Hcy levels between children with excess fat mass and healthy controls, as well as no correlations between Hcy and glucose or lipid parameters, nor with indices of IR [23]. Our findings are consistent with these latter studies. Similarly, Santos et al. [24] found no statistically significant differences in Hcy levels among overweight, obese, and severely obese children, and no associations with IR, BP, or renal function. Interestingly, Gallistl et al. [48] reported that changes in tHcy levels among 56 obese children were independently and significantly associated with baseline lean body mass (LBM) and were inversely related to changes in LBM. The authors emphasized that weight reduction in obese children should aim not only to decrease total body weight but also to preserve or increase lean body mass.

The prevalence of obesity has reached pandemic proportions, contributing not only to metabolic disorders such as IR and dyslipidemia but also to vascular dysfunction through mechanisms involving chronic inflammation and oxidative stress. As demonstrated in previously cited studies, the relationship between Hcy levels and the lipid profile remains inconsistent, with some studies reporting no association [21,23,25,26] and others identifying a positive correlation [18,27]. Similarly, findings regarding the relationship between Hcy and IR are equivocal. Some studies [18,20] found no link between Hcy levels and insulin status or HOMA-IR; others [22,26,28] reported a significant association. Martos et al. [26], in a case–control study, observed elevated Hcy levels exclusively in obese hyperinsulinemic children compared to their normoinsulinemic obese peers. In these obese children, Hcy positively correlated with insulin, HOMA-IR, and leptin, as well as with CRP and IL-6. However, multivariate regression analysis identified only HOMA-IR and leptin as independent predictors of Hcy levels. In our study, we did not observe an association between tHcy and IR or atherogenic indices. Given the pro-inflammatory role attributed to Hcy, we also investigated this aspect by assessing leukocyte profiles and CRP concentrations. However, our analysis did not reveal any significant relationship between tHcy and inflammatory markers.

A recent prospective study [42] investigated the association between Hcy levels and CVD risk factors in a large cohort of 2102 adolescents aged 14 to 19 years. Although positive associations were observed between Hcy and age, creatinine, BMI, and BP, and negative correlations with glomerular filtration rate (GFR), HDL-C, glycated hemoglobin (HbA1c), insulin, and HOMA-IR, multivariate analysis revealed sex and creatinine as the strongest predictors of Hcy levels. Consistent with these findings, we observed a correlation between tHcy and creatinine levels. Patients with CKD typically have elevated Hcy levels, which can be three- to fivefold higher than normal in those with end-stage renal disease (ESRD) [12]. Since Hcy is primarily metabolized via the transsulfuration pathway in the kidneys, impaired renal function may lead to increased plasma Hcy levels. Moreover, Hcy may contribute to kidney damage through mechanisms involving ROS formation, induction of local oxidative stress, inflammation, endoplasmic reticulum stress, and vascular injury by DNA hypomethylation [12].

Homocysteine levels are known to be influenced by genetic factors. The TT genotype of the MTHFR C677T polymorphism, for example, has been associated with increased risk of obesity and elevated Hcy levels in obese individuals [6]. In a pediatric cohort comprising 128 adolescents and 195 children, age was identified as a major determinant of Hcy levels, independent of biochemical parameters (e.g., lipid profile, creatinine, uric acid), Hcy-related metabolic markers (e.g., folate, vitamin B12, holotranscobalamin, methylmalonic acid), and genetic variants, including MTHFR 688C>T [49]. Similarly, Gara et al. [50] found no significant differences in tHcy, folate, vitamin B12 levels, or in the allelic distribution of the C677T and G80A polymorphisms in the MTHFR and RFC genes between obese and non-obese Tunisian children. In our study, which primarily focuses on anthropometric and biochemical parameters, we also identified age as an independent factor influencing tHcy levels.

The present study has some limitations. First, we did not directly assess vitamin B12 and folate levels, which are involved in the Hcy metabolism. However, we evaluated the erythropoietic profile of all participants, which may indirectly reflect deficiencies in these vitamins. Additionally, a detailed dietary analysis of vitamins was not performed, although all children followed an unrestricted diet, making a significant influence on Hcy levels unlikely. Another limitation is the relatively small sample size, which may have impacted the reliability and statistical power of our results. Finally, genetic polymorphisms in enzymes involved in Hcy metabolism were not evaluated.

## 5. Conclusions

In this study, no significant differences in plasma tHcy levels were observed among obese, overweight, and normal-weight adolescents. Furthermore, tHcy levels were not associated with typical cardiometabolic risk factors in obese children. Age was identified as the most substantial independent predictor of tHcy levels. The observed association with creatinine suggests a possible renal contribution to Hcy metabolism.

Our study suggests that routine tHcy screening based solely on obesity status in otherwise healthy adolescents may not be warranted. However, age and renal function appear to be important contributing factors.

## Figures and Tables

**Table 1:** The comparison of anthropometric measurements, tHcy levels, and biochemical parameters between overweight children, obese children, and their normal-weight peers.

Variable	Control Children (n = 25)	Overweight Children (n = 14)	Obese Children (n = 42)
**Age** (years)	14.98 ± 1.84	13.00 ± 2.21 ^#^	13.86 ± 2.19 *
**Height** (cm)	163.66 ± 10.96	159.54 ± 13.13	166.26 ± 9.76
**Body weight** (kg)	55.50 (45.70–61.60)	61.30 (54.20–70.60)	89.85 (75.80–98.40) *^
**BMI** (kg/m^2^)	20.20 (17.80–22.50)	24.55 (24.10–27.30) **^#^**	30.75 (29.10–33.70) *^
**BMI SDS**	0.00 (−0.60–0.80)	1.55 (1.40–1.80) **^#^**	2.30 (2.10–2.60) *^
**WC** (cm)	65.43 ± 5.92	81.75 ± 6.24 **^#^**	92.50 ± 7.63 *^
**HC** (cm)	85.57 ± 9.45	94.42 ± 9.81^#^	109.46 ± 9.27 *^
**WHR**	0.78 (0.75–0.80)	0.86 (0.82–0.90) **^#^**	0.84 (0.80–0.90) *
**WHtR**	0.41 ± 0.03	0.51 ± 0.04 **^#^**	0.55 ± 0.04 *^
**% BFM**	23.89 ± 7.03	31.62 ± 3.38 **^#^**	37.39 ± 5.61 *^
**tHcy** (µmol/L)	12.45 (10.88–14.44)	10.71 (9.79–13.50)	11.09 (9.04–12.81)
**Glucose** (mg/dL)	83.08 ± 7.25	85 ± 7.22	86.63 ± 6.53
**Insulin** (µIU/mL)	8.67 (2.72–11.20)	12.74 (9.48–21.00)	15.50 (10.20–22.90) *
**FGIR**	9.24 (6.94–34.93)	6.60 (4.56–9.21)	5.85 (3.97–8.56) *
**HOMA-IR**	1.79 (0.94–2.55)	2.56 (1.97–4.77)	3.17 (2.13–4.98) *
**QUICKI**	0.35 (0.34–0.41)	0.33 (0.30–0.34)	0.32 (0.30–0.34) *
**MATSUDA**	-	3.15 (2.54–4.38)	2.58 (2.00–3.70)
**TC** (mg/dL)	148.48 ± 21.12	166.14 ± 22.49	163.07 ± 26.62
**HDL-C** (mg/dL)	58.68 ± 11.98	51.50 ± 12.26	44.07 ± 11.23 *
**LDL-C** (mg/dL)	77.45 ± 19.84	95.06 ± 21.40	93.92 ± 25.48
**TG** (mg/dL)	60.00 (43.00–69.00)	97.93 (79.00–105.00)	108.00 (93.00–157.00) *
**TG/HDL-C**	1.09 (0.72–1.36)	1.79 (1.41–2.32) **^#^**	2.35 (1.66–4.16) *
**non HDL**	93.00 (72.00–106.00)	115.50 (98.00–131.00)	115.00 (99.00–133.00)
**CRP** (mg/dL)	0.50 (0.50–0.50)	0.50 (0.50–0.50)	0.50 (0.50–0.65)
**Creatinine** (mg/dL)	0.70 (0.60–0.70)	0.60 (0.50–0.70)	0.60 (0.50–0.60)
**WBC** (cells × 10^3^/µL)	6.21 ± 1.74	6.21 ± 1.52	6.94 ± 1.42
**Neutrophil** (cells × 10^3^/µL)	3.02 ± 1.40	3.06 ± 1.02	3.73 ± 1.13
**Lymphocyte** (cells × 10^3^/µL)	2.46 ± 0.70	2.29 ± 0.64	2.39 ± 0.50
**Monocyte** (cells × 10^3^/µL)	0.46 (0.42–0.56)	0.55 (0.49–0.60)	0.51 (0.48–0.63)
**Eosinophil** (cells × 10^3^/µL)	0.16 (0.11–0.30)	0.20 (0.16–0.36)	0.20 (0.10–0.30)
**Basophil** (cells × 10^3^/µL)	0.03 (0.02–0.05)	0.01 (0.00–0.03)	0.02 (0.00–0.03) *
**SBP** (mmHg)	110.09 ± 12.47	116.83 ± 9.24	124.42 ± 5.50 *
**DBP** (mmHg)	70.00 (63.00–76.00)	75.00 (63.00–78.00)	74.00(67.00–77.00)

Data are presented as mean ± standard deviation (SD) or median values with 25th–75th percentiles, as appropriate. * *p* < 0.05—compared obese children to normal-weight children. ^#^ *p* < 0.05—compared overweight children to normal-weight children. ^ *p* < 0.05—compared obese children to overweight children.

**Table 2:** Comparison of anthropometric measurements, tHcy levels, and carbohydrate and lipid parameters between girls and boys with overweight and obesity.

Variable	Girls (n = 27)	Boys (n = 29)
**Age** (years)	14.33 ± 2.22	13.00 ± 2.14
**Height** (cm)	163.78 ± 7.85	165.33 ± 13.35
**Body Weight** (kg)	83.61 ± 16.29	80.33 ± 18.99
**BMI** (kg/m^2^)	30.30 (27.30–34.40)	29.70 (26.60–30.90)
**BMI SDS**	2.40 (2.00–2.70)	2.10 (1.8–2.30) *
**WC** (cm)	88.48 ± 8.76	91.04 ± 8.51
**HC** (cm)	109.74 ± 10.81	101.98 ± 10.83 *
**WHR**	0.81 (0.78–0.83)	0.89 (0.84–0.93) *
**WHtR**	0.54 ± 0.05	0.55 ± 0.05
**% BFM**	37.54 ± 4.38	40.64 ± 4.93 (0.062)
**tHcy** (µmol/L)	10.34 (8.59–12.74)	11.26 (9.79–13.89)
**Glucose** (mg/dL)	86.17 ± 6.59	86.57 ± 6.82
**Insulin** (µIU/mL)	15.50 (9.97–22.90)	13.65 (9.18–21.45)
**FGIR**	5.38 (3.97–8.56)	6.44 (4.25–9.60)
**HOMA-IR**	3.17 (2.04–5.15)	2.81 (1.99–4.88)
**QUICKI**	0.32 (0.30–0.34)	0.33 (0.30–0.34)
**MATSUDA**	2.56 (2.01–4.30)	2.95 (1.91–3.93)
**TC** (mg/dL)	164.78 ± 28.15	162.96 ± 23.21
**HDL-C** (mg/dL)	46.41 ± 12.46	45.48 ± 11.43
**LDL-C** (mg/dL)	92.44 ± 25.93	95.85 ± 23.10
**TG** (mg/dL)	116.00 (97.00–168.00)	95.20 (79.00–115.00) *
**TG/HDL-C**	2.31 (1.51–4.16)	2.06 (1.57–3.12)
**non HDL**	115.00 (98.00–138.00)	115.00 (105.00–131.00)

Data are presented as mean ± standard deviation (SD) or median values with 25th–75th percentiles, as appropriate. * *p* < 0.05 compared girls to boys.

## Data Availability

Dataset available on request from the authors.

## Abbreviations

Hcy	Homocysteine
tHcy	Total Homocysteine
HHcy	Hyperhomocysteinemia
ROS	Reactive oxygen species
NO	Nitric oxide
DNA	Deoxyribonucleic acid
RNA	Ribonucleic acid
CVD	Cardiovascular disorders
IR	Insulin resistance
BMI	Body Mass Index
SDS	Standard Deviation Score
MCV	Mean corpuscular volume
WC	Waist circumference,
HC	Hip circumference,
WHR	Waist-to-hip ratio
WHtR	Waist-to-height ratio
BFM	Body fat mass
TC	Total cholesterol
HDL-C	High-density lipoprotein cholesterol
TG	Triglycerides
LDL-C	Low-density lipoprotein cholesterol
CRP	C-reactive protein
OGTT	Oral glucose tolerance test (OGTT)
FGIR	Fasting glucose-to-insulin ratio
QUICKI	Quantitative Insulin Sensitivity Check Index
HOMA-IR	Homeostasis Model Assessment-Insulin Resistance
TG/HDL-C	Triglyceride-to-high-density lipoprotein cholesterol ratio
BP	Blood pressure
WBC	White Blood Cells
SBP	Systolic blood pressure
DBP	Diastolic blood pressure
AMPK	Adenosine monophosphate-activated protein kinase
IRS-1	Insulin receptor substrate-1
PI3K/Akt	Phosphoinositide-3-kinase/protein kinase B
MAPK	Mitogen-activated protein kinase
NF-κB	Nuclear factor kappa-light-chain-enhancer of activated B cells
IL	Interleukin
TNF-α	Tumor necrosis factor alpha
CBS	Cystathionine β-synthase
5-MTHF	5-methyltetrahydrofolate
THF	Tetrahydrofolate
DMG	N,N-dimethylglycine
LBM	Baseline lean body mass
GFR	Glomerular Filtration Rate
HbA1c	Glycated hemoglobin
CKD	Chronic kidney disease
ESRD	End-stage renal disease
